# Targeting Nuclear Factor-Kappa B to Overcome Resistance to Chemotherapy

**DOI:** 10.3389/fonc.2013.00120

**Published:** 2013-05-16

**Authors:** P. Godwin, A. M. Baird, S. Heavey, M. P. Barr, K. J. O’Byrne, K. Gately

**Affiliations:** ^1^Department of Clinical Medicine, Thoracic Oncology Research Group, Trinity College Dublin, St. James’s Hospital IrelandDublin, Ireland

**Keywords:** NF-κB, cancer, cisplatin, chemotherapy, resistance, apoptosis, oncogene

## Abstract

Intrinsic or acquired resistance to chemotherapeutic agents is a common phenomenon and a major challenge in the treatment of cancer patients. Chemoresistance is defined by a complex network of factors including multi-drug resistance proteins, reduced cellular uptake of the drug, enhanced DNA repair, intracellular drug inactivation, and evasion of apoptosis. Pre-clinical models have demonstrated that many chemotherapy drugs, such as platinum-based agents, antracyclines, and taxanes, promote the activation of the NF-κB pathway. NF-κB is a key transcription factor, playing a role in the development and progression of cancer and chemoresistance through the activation of a multitude of mediators including anti-apoptotic genes. Consequently, NF-κB has emerged as a promising anti-cancer target. Here, we describe the role of NF-κB in cancer and in the development of resistance, particularly cisplatin. Additionally, the potential benefits and disadvantages of targeting NF-κB signaling by pharmacological intervention will be addressed.

## Introduction

Platinum-based anti-cancer drugs, such as cisplatin, play a crucial role in the treatment of cancer. The cytotoxic effect of these drugs relies on their ability to induce DNA damage. However, intrinsic or acquired resistance to chemotherapy often critically limits the efficacy and outcome of treatment. Much progress has been made delineating the mechanisms of cellular resistance, which include reduced intracellular accumulation of the drug, increased DNA repair and altered oncogene and regulatory protein expression. In addition, increased expression of anti-apoptotic genes and mutations in the intrinsic apoptotic pathway contribute to impaired DNA damage detection and apoptosis induction (Kartalou and Essigmann, 2001). Recently, Nuclear Factor-kappa B (NF-κB) has been identified as a key player in resistance mechanisms (Thevenod et al., 2000; Bottero et al., 2001; Braeuer et al., 2006).

Nuclear Factor-kappa B (NF-κB) is a tightly regulated transcription factor, composed of homo or heterodimers from a pool of five REL proteins: NF-κB1(p50), NF-κB2 (p52), RelA (p65), RelB, and c-Rel (Rel). However, their biological effects are cell type dependant, mediating diverse physiological processes. In un-stimulated cells, NF-κB is sequestered within the cytoplasm, bound to its regulatory protein, inhibitor of NF-κB (IκB). NF-κB activation is driven by the phosphorylation of IκB, resulting in the dissociation of the NF-κB:IκB complex. Consequently, exposing the nuclear localization sequence of NF-κB, thus inducing its translocation into the nucleus (Ghosh et al., 1998). After homo/heterodimer formation, NF-κB binds to specific promoter sequences (κB sites) contained within many genes, which play important roles in cellular growth and apoptosis (Pahl, 1999).

**Figure 1:**
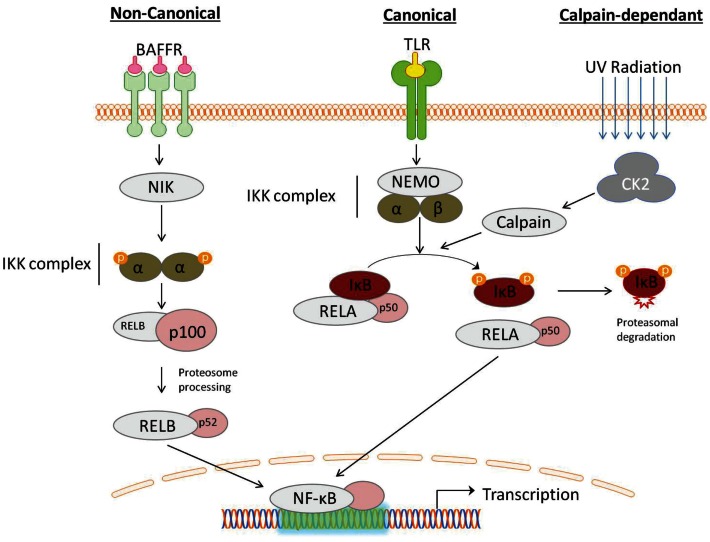
**Activation of NF-κB can be described by distinct pathways, according to the mechanism of IκB degradation**. Canonical signaling, initiated by toll-like-receptor (TLR) ligation, is dependent on the IKK complex containing NF-κB essential modulator (NEMO) and IKKα/IKKβ. Non-canonical signaling occurs in response to ligation of a subset of tumor-necrosis factor receptor (TNFs) members, is dependent on the NF-κB-inducing kinase (NIK), which activates proteolytic processing of p100 by IKKα. NF- κB activation can also occur through a IKK-independent mechanism, induced by DNA damage detection, involving the serine threonine kinase casein kinase 2 (CK2), which activates the IκB targeting cysteine protease calpain.

Traditionally the activation of NF-κB occurs through two independent signaling pathways: in the canonical pathway, binding of a ligand to a cell surface receptor, such as a member of the toll-like-receptor (TLR) superfamily, leads to the recruitment of adaptors (such as TRAF) to the cytoplasmic domain. TRAF recruits and activates the IKK complex, containing the IKKβ or IKKα protein and the scaffold protein, NF-κB essential modulator (NEMO). IKK phosphorylates two serine residues (ser^32^/ser^36^), in the IκBα regulatory domain resulting in dissociation of the NFκB:IκB complex. The non-canonical pathway is activated in response to non-inflammatory stimuli, such as BAFFR induced B-cell maturation (Crowley et al., 2005). Non-canonical signaling is mediated via IKKα independent of NEMO, but requires NF-κB-inducing kinase (NIK) (Li and Verma, 2002). Recently, an alternate pathway of NF-κB activation has also been described, which does not require IKK. For example, the activation of NF-κB by UV light occurs via casein kinase 2 and subsequent calpain-dependent IκB degradation (Figure 1) (Kato et al., 2003).

## NF-κB and Cancer

NF-κB and its target genes have been implicated as mediators in all of the hallmarks of cancer (Pahl, 1999; Hanahan and Weinberg, 2011). Constitutive activation of NF-κB has been demonstrated in lung (Tew et al., 2008), breast (Chua et al., 2007), lymphoma (Zou et al., 2007), and leukemia (Vilimas et al., 2007) cell lines. Elevated NF-κB levels correlate with poor prognostic outcome in ovarian cancer (Annunziata et al., 2010) and glioblastoma (Brown and Law, 2006). The inhibition of NF-κB signaling or NF-κB gene knockout, has been shown to mediate anti-tumor responses (Li et al., 2005a; Meylan et al., 2009).

## NF-κB and Chemotherapeutic Resistance

In addition to its role in cancer cell survival (Li et al., 2005a), activated NF-κB has also been identified as a key mechanism of cisplatin resistance. NF-κB activity inversely correlated with cellular sensitivity to chemotherapy in carcinoma cell lines (Chuang et al., 2002). Furthermore, long term treatment of a panel of lung cancer cell lines, with increasing doses of cisplatin, produced cell lines resistant to cisplatin mediated cell death (Barr et al., 2013). These resistant cells show increased expression of NF-κB, compared to their matched cisplatin sensitive parent, implicating NF-κB as a potential mediator of acquired cisplatin resistance (unpublished data).

Nuclear proteins, isolated from prostate cancer cells treated with cisplatin, had activated NF-κB levels greater than untreated control cells, as measured by increased DNA binding activity. This activity could be abrogated by transfection with p65 siRNA. Cisplatin, therefore, enhanced the DNA binding activity of NF-κB, through increased expression and activation of the protein, thereby limiting its own potential efficacy. Treatment of cells with genistein, an AKT-NF-κB inhibitor, abrogated the increased NF-κB activity in murine models, sensitizing them to cisplatin induced apoptosis (Li et al., 2005a).

### NF-κB signaling

Many cancer cells rely on oncogene addiction for maintenance of their malignant phenotype (Weinstein, 2002). This over reliance can dictate the choice of molecularly targeted therapies, as inhibition of these “addictive” pathways, such as the NF-κB pathway, can sensitize cancer cells to apoptosis (Ariga et al., 2002). However, the activation of NF-κB is inherently complex, given the multitude of signaling pathways and crosstalk connections which can stimulate it. It is therefore essential to identify cancer cell specific pathways, to broaden our understanding of NF-κB signaling, thus allowing for rational selection of suitable inhibitors.

#### Growth factor receptor stimulation

In response to extracellular signals, a number of growth factor receptors can activate NF-κB via the PI3K/AKT pathway (Crowley et al., 2005). Elevated expression of EGFR in ovarian, cervical, bladder, and esophageal cancer has been correlated with poor prognosis in the clinical setting (Nicholson et al., 2001). Cisplatin has been shown to possess EGF-like activity, as treatment of A549 cells with cisplatin resulted in the phosphorylation of EGFR and the activation of the PI3K/AKT/NF-κB pathway. Therefore, in NSCLC a potential explanation for cisplatin induced NF-κB expression may occur through the stimulation of EGFR (Kuroda et al., 2010). This data suggests that treatment with cisplatin may induce constitutive activation of EGFR, and consequently cisplatin resistance via NF-κB.

#### The PI3K/AKT pathway

The PI3K/AKT pathway responds to a milieu of intracellular and extracellular signals via receptor tyrosine kinase (RTK) receptors. AKT is a critical signaling molecule, which acts as a central hub for many cell growth and survival pathway. Activating PI3K pathway mutations are common in cancer and have been implicated in the development of cisplatin resistance. PI3K/AKT inhibitors re-sensitize cells to treatment in many cancer types, including ovarian (Non-aka et al., 2012). Primary acute myeloid leukemia cells, with constitutive AKT activation, treated with the AKT inhibitor LY294002, showed a reduction in both p-AKT, and NF-κB levels resulting in apoptosis. In this instance NF-κB activation is occurring in an AKT-dependent manner. Therefore, the AKT-NF-kB pathway may represent a viable mechanism for overactive PI3-kinase to promote resistance to cytotoxic agents (Grandage et al., 2005).

AKT mediated NF-κB activation requires the effector molecule mTOR, which forms the catalytic subunit of two molecular signaling complexes; mTOR complex 1 (mTORC1) and 2 (mTORC2). mTORC1 is associated with endosomal membranes, where it promotes the translation of NF-κB regulated mRNAs, such as Bcl-2, Bcl-xL, and cyclin D, through the effector 4E-binding protein 1 (4E-BP1) (De Benedetti and Graff, 2004). Chronic activation of mTORC1 is frequent in cancer (Meric-Bernstam and Gonzalez-Angulo, 2009). AKT dependant mTORC1 activation occurs through phosphorylation of the tuberous sclerosis complex (TSC) and subsequent activation of the mTORC1 regulator, Rheb (Inoki et al., 2002). Rapamycin induced inhibition of mTORC1 suppresses breast cancer induction in mouse models (Namba et al., 2006), whereas incremental Rheb induction promotes metastasis and tumor progression (Lu et al., 2010). Therefore, PI3K/AKT-dependent mTORC1 activation contributes to cancer promotion and chemoresistance possibly through the translation of NF-κB target genes.

In contrast, mTORC2 may be activated through ribosome binding and once activated phosphorylates AKT ser^473^ (Sarbassov et al., 2005; Zinzalla et al., 2011). It’s role in cancer is not clear, although it is required for PTEN loss-induced prostate cancer in mice, suggesting a central role in PI3K-dependant carcinogenesis (Guertin and Sabatini, 2009). The allosteric mTOR inhibitor, rapamycin, does not inhibit mTORC2, offering an explanation for the failure of rapamycin in PI3K-hyperactivated cancer (Cloughesy et al., 2008). PI3K signaling is hyperactivated in 90% of glioblastomas, and is associated with EGFRvIII, a mutant activated EGFR, and mTORC2 activation. In addition, EGFRvIII mutants express elevated NF-κB activity and resistance to DNA damaging agents. These mutants are dependent on mTORC2 expression but not AKT or mTORC1 signaling (Tanaka et al., 2011). mTORC2 represents a mechanism of NF-κB activation and subsequent chemotherapeutic resistance in EGFR mutated cancers.

#### The MAP kinase/ERK pathway

The RAS-Mitogen activated Protein Kinase (MAPK) pathway is integral to transducing extracellular signals, including cytokines and growth factors (Enzler et al., 2011). Central to this pathway is the activation of RAS by the guanine exchange factor son of sevenless (SOS). The activated GTP-bound RAS activates the serine/threonine kinase, RAF, which in turn activates MAPKs, including extracellular signal regulated kinase (ERK). ERK 1/2 translocate to the nucleus where they activate the Jun/Fos family of transcription factors. Due to its central role in cellular proliferation it is not surprising that the SOS-Ras-Raf-MAPK cascade is often mutated in cancer, with RAS mutations found in ∼45% of colon and ∼90% of pancreatic cancers (Katz et al., 2007).

Cisplatin induces the persistent activation of the apoptotic MAPK and c-jun N-terminal kinase (JNK) pathways. The apoptotic nature of this pathway may be through MAP2K and MEKK1, which activate JNK. This pathway may also activate NF-κB, resulting in differential apoptotic signaling. Through this mechanism MAPK can drive the activation of NF-κB in response to cisplatin treatment (Sanchez-Perez et al., 2002). This means that inactivating mutations in the JNK pathway could lead to signaling only via the NF-κB pathway in response to cisplatin treatment, thereby providing a potential mechanism of resistance.

Raf kinase inhibitor protein (RKIP) is a metastasis suppressor/immunosurveillance cancer gene, which acts as a negative regulator of both the MAPK cascade, initiated by Raf-1 (Yeung et al., 1999), and NF-κB activation, through the negative regulation of IκB (Yeung et al., 2001). RKIP loss or depletion has been correlated with metastasis and chemotherapeutic resistance in solid tumors (Chatterjee et al., 2004). In prostate cancer, RKIP is regulated by the transcriptional repressor Snail. Transcription of Snail is partly regulated by NF-κB (Julien et al., 2007) and is a key modulator in metastasis (Beach et al., 2008). Prostate cancer cells which exhibit constitutive NF-κB activation express increased levels of Snail, which in turn inhibits RKIP expression protecting cells from chemotherapy induced apoptosis (Baritaki et al., 2009). Inhibition of NF-κB, by DHMEQ, decreases the expression of Snail and Bcl-xL leading to elevated expression levels of RKIP, thereby re-sensitizing cells to chemotherapy (Baritaki et al., 2009).

#### Janus Kinase/Signal Transducers and Activators of Transcription pathway

Upon binding to their cognate receptors, receptor associated Janus Kinases (JAKs) phosphorylate ligand-bound receptors resulting in the recruitment and activation of Signal Transducers and Activators of Transcription (STATs) (Darnell et al., 1994). Phosphorylated STATs form homodimers, localize to the nucleus, and regulate genes associated with apoptosis and proliferation (Darnell, 2002). Persistent activation of STATs has been shown in primary tumors and cancer cell lines, where it is believed to contribute to oncogenesis (Bromberg, 2002). It is thought that STAT3 can bind to and cause the dissociation of the NF-κB:IκB complex (Yang et al., 2007). This can prolong NF-κB retention in the nucleus by altering its acetylation status via interactions with p300 (Lee et al., 2009). STAT3 can therefore promote NF-κB activation even in the absence of IKK signaling. Silencing of STAT3 reduced Bcl-xL expression, a NF-κB target gene, re-sensitizing A549 cells to cisplatin (Kulesza et al., 2013).

### Oncogenes and NF-κB

p53 is a tumor suppressor protein activated upon cellular stress, such as DNA damage. Once activated it binds DNA, where it modulates the expression of genes associated with DNA repair and apoptosis (MacFarlane et al., 1996). p53 is therefore an important mediator of cisplatin induced cell death. Interestingly, NF-κB can bind the p53 promoter region inducing its transcription (Kim et al., 2002), to form a negative feedback loop. Loss of function p53 mutations are a common occurrence in cancer (Greenblatt et al., 1994) and the failure of this control mechanism could potentially induce constitutive activation of the NF-κB pathway. A panel of p53 mutated NSCLC cell lines, which were resistant to cisplatin, became sensitive to treatment upon transfection with the p53 wild type gene (Lai et al., 2000). Thus, p53 gene status may play a significant role in the development of cisplatin resistant NSCLC.

The impact of a p53 mutation in NSCLC can be influenced by the presence of additional oncogenic mutations. The signaling protein, Ras, acts as molecular switch and once activated it propagates downstream signaling pathways leading to NF-κB activation via the IKK tank-binding kinase 1 (TBK1) (Barbie et al., 2009). NF-κB activation by oncogenic Ras (KRAS) is implicated in lung carcinogenesis (Barbie et al., 2009). KRAS mutations are found in 20–30% of NSCLC cases, while inactivating mutations of p53 occur at a rate of approximately 50% (Herbst et al., 2008). Furthermore, both mutations when occurring simultaneously lead to the development of lung adenocarcinoma in mouse models, which may be as a result of increased NFκB activity (Meylan et al., 2009). Cisplatin induced apoptosis is likely to be mediated, at least in part, by the induction of p53 in response to DNA damage. Consequently, resistance to cisplatin in some cancers may be mediated through KRAS mediated NF-κB activation.

### NF-κB and DNA repair

Elimination of DNA damage, by cellular repair mechanisms or destruction of the cell via apoptosis, is essential for the maintenance of genome integrity and disease prevention. It is known that the threshold for the induction of apoptosis is lower in cancer cells in response to DNA damage compared with their normal counterparts (Evan and Vousden, 2001; Enzler et al., 2011). Platinum-based drugs induce apoptosis through DNA adduct formation (Chaney et al., 2004). In mouse models, it has been shown that cisplatin resistant cancer cells repair DNA adducts more efficiently, compared with sensitive cells (Oliver et al., 2010). Nucleotide excision repair (NER), a major DNA repair pathway, repairs bulk DNA damaged by environmental factors and chemotherapy (Friedberg et al., 2006). The NER rate-limiting step is affected by the protein products of the genes ERCC1 and XPAC. Up-regulated expression of these genes has been correlated with increased drug resistance in ovarian cancer (Dabholkar et al., 1994).

In a study of the role of Bcl-xL in NER-facilitated protection against cisplatin, it was demonstrated that NER was required for cisplatin induced NF-κB translocation. The exact mechanism through which NER activates NF-κB is unknown, but is understood to be dependent on the ataxia telangiectasia mutated (ATM) protein (Wuerzberger-Davis et al., 2007). The ATM-NF-κB pathway is believed to be negatively regulated by ATM and rad-3-related (ATR) kinase. ATR has been shown to repress NF-κB activation in response to replicative stress, by competitively binding to NEMO (Wu and Miyamoto, 2008). Consequently cells with high levels of ATR activity show decreased NF-κB levels and increased apoptosis in response to cisplatin (Lomonaco et al., 2009).

### NF-κB target genes and apoptosis

NF-κB target genes play an important role in the regulation of many of the pathways involved in the hallmarks of cancer. Genes regulated by NF-κB include those that are associated with inflammation (TNF, IL-6, and ICAM), cell survival (cIAP1/2, Bcl-2, and Bcl-xL), proliferation (CDK2), tumor progression (COX2), angiogenesis (VEGF), and also cell death (Fas and FasL) (Pahl, 1999). Apoptosis can be initiated by signals which occur outside or within the cell. These signals may promote cell survival, such as growth factor stimulation, or induce apoptosis, e.g., Tumor-necrosis factor (TNF). The apoptotic pathway is described through two distinct mechanisms; loss of mitochondrial function and death receptor signaling through the activation of the caspase cascade. It is the fine balance of these antagonistic signals which ultimately determines cell fate.

#### Mitochondrial dysregulation

The mitochondrial pathway of caspase activation is controlled by the Bcl-2 family proteins, which act by modulating the release of caspase activators from mitochondria (Estaquier et al., 2012). In a prostate cancer cell line, PC3, the levels of anti-apoptotic signaling proteins Bcl-2, Bcl-xL, and survivin were decreased in response to the NF-κB inhibitor genistein. The decrease in expression of these proteins occurred in association with the up-regulation of p21^waf1^, a pro-apoptotic protein and cell cycle inhibitor. Genistein re-sensitized resistant cells to docetaxel and cisplatin mediated apoptosis (Li et al., 2005a). Similarly in the aggressive cisplatin resistant cervical cancer cell line, SKOV3.ip1, inhibition of NF-κB led to increased expression of Bid and decreased Bcl-2 and Bcl-xL levels. This shift in expression of pro- and anti-apoptotic proteins was reflected in the increased sensitivity of these cells to cisplatin treatment (Yang et al., 2011). Apoptotic deregulation may represent an important mechanism through which NF-κB mediates resistance.

#### Death receptor signaling

Members of the TNF receptor (TNFR) family and their corresponding ligands are crucial regulators of various cellular processes. A number of TNFRs, including Fas and TRAIL, are potent activators of the apoptotic caspase cascade through adaptor molecules such as FADD (Ashkenazi and Dixit, 1998). Due to their role in the regulation of apoptosis they are often referred to as death receptors (DRs). Cell death induced by these DRs is tightly regulated by NF-κB target genes, including inhibitor of apoptosis proteins (IAPs), TRAF-1/2 (Wang et al., 1998), and Bcl-2 proteins (Wang et al., 1999). However, it has been shown that ectopic over-expression of these genes affords only partial protection from DR mediated apoptosis (Wang et al., 1999). More recently, a potent inhibitor of DR signaling has been identified, called c-FLIP.

Micheau et al. (1999) first alluded to the role of c-FLIP in chemoresistance in a study which demonstrated that the inhibition of the FADD-caspase 8 pathway resulted in a decrease in tumor cell sensitivity to drug-induced apoptosis. In a study to identify novel pro-apoptotic proteins up-regulated by NF-κB-inducing ligands (CD40, IL-1, and TNF), c-FLIP expression was found to be increased at both mRNA and protein level. Analysis of Jurkat cell lines, including an IκB over-expressing and NEMO deficient mutant, treated with the NF-κB-inducing ligand P/I, showed abrogated c-FLIP expression compared to wild type control, indicating c-FLIP expression was mediated by NF-κB (Kreuz et al., 2001). The murine B-cell line A20, susceptible to Fas mediated apoptosis, was protected from apoptosis through B-cell receptor (CD40) signaling, which is known to activate NF-κB. Pre-treatment of B-cells with CD40 increased c-FLIP expression, mediating resistance to a subsequent challenge with FasL. Conversely, dendritic cells resistant to FasL could be sensitized to apoptosis by pre-treating with cycloheximide. Cycloheximide prevents proteosomal degradation of IκB, thereby preventing NF-κB activation and subsequent c-FLIP expression (Micheau et al., 2001). Similarly, it was shown that cisplatin-selected cervix carcinoma HeLa cell lines induced less apoptosis in response to treatment with cisplatin or Fas-activating antibody, than parental cells. Negative regulator of apoptosis c-FLIP, but not Bcl-2, Bcl-xL, or IAPs, was found to be higher in resistant cells. Treatment with c-FLIP anti-sense oligonucleotides promotes cisplatin and Fas-antibody-induced apoptosis to a much greater degree in resistant cells (Kamarajan et al., 2003). The cancer associated gene (CAGE) regulates expression of epithelial-mesenchymal transition (EMT)-related proteins through ERK, AKT, and NF-κB in mouse B16F10 melanoma cells. Snail and c-FLIP mediate the effects of CAGE protecting cells against celastrol, an anti-cancer agent (Kim et al., 2009).

In ovarian cancer cells the expression of c-FLIP, in chemosensitive (OV2008) and chemoresistant (C13*) cells treated with cisplatin and/or transfected with c-FLIP siRNA or cDNA, was measured. Treatment with cisplatin significantly decreased c-FLIP levels in sensitive but not resistance cell lines. In OV2008 cells, c-FLIP over-expression attenuated apoptosis, while the down regulation of c-FLIP in C13* cells increased cisplatin mediated apoptosis (Abedini et al., 2004). Furthermore, in cisplatin sensitive (T24) and resistant (T24R2) bladder cancer cell lines, cisplatin mediated suppression of c-FLIP expression was correlated with sensitivity (Lee et al., 2013). Downregulation of c-FLIP expression has been shown to sensitize many cancer types to chemotherapy including cervical adenocarcinoma (Luo et al., 2008), breast (Rogers et al., 2007), colorectal (Longley et al., 2006), and prostate (Wilson et al., 2008). Cisplatin mediated c-FLIP degradation has been proposed to occur via p53 dependant ubiquitination and proteasomal degradation of c-FLIP (Abedini et al., 2008). This indicates a possible mechanism of cisplatin resistance whereby loss or dysregulation of p53 results in the loss of cisplatin mediated c-FLIP degradation and failure to induce apoptosis.

#### Caspase cascade

Inhibitor of apoptosis proteins are a family of potent apoptosis inhibitors characterized by three baculoviral IAPs that bind to and inhibit caspase activity (Fu et al., 2003). In colorectal cancers, the X-linked IAP (XIAP) mRNA levels are significantly higher in primary cancer tissue compared with normal mucosa, and expression was correlated with disease progression and metastasis (Takeuchi et al., 2005). Furthermore, in the cisplatin resistant prostate cell line, LNCaP, IAP expression increased with the extent of cisplatin resistance (Nomura et al., 2005). In a study of TRAIL induced apoptosis it was found that constitutive activation of NF-κB led to increased XIAP expression and resistance to TRAIL mediated cell death (Braeuer et al., 2006). Similarly in melanoma cell lines, the inhibition of NF-κB resulted in decreased XIAP expression and induction of apoptosis (Bush et al., 2001). Furthermore, in melanoma cells depletion of endogenous XIAP promoted apoptosis and reduced clonogenicity of cancer cells treated with cisplatin (Silke et al., 2005). In a clinical study of head and neck squamous cell carcinoma, high XIAP levels correlated with increased resistance to cisplatin and decreased survival (Yang et al., 2012). The expression levels of XIAP represent an important factor in determining cell fate in response to apoptotic signaling and cisplatin.

## Pro-Apoptotic Role of NF-κB

Induction of NF-κB in response to chemotherapy often leads to a dysregulated apoptotic response, which may mediate chemoresistance. Consequently, much work has focused on the development of targeted therapies that block the NF-κB pathway in order to induce cell death. However, due to the complexity of NF-κB signaling, both pro- and anti-tumourigenic activities may result (Lin et al., 1999; reviewed in Pikarsky and Ben-Neriah, 2006). It is likely that several factors including cell type, nature of stimulus and chromatin modifications can confer pro or anti-apoptotic activities of NF-κB (Radhakrishnan and Kamalakaran, 2006; Barisic et al., 2008).

The most simplistic mechanism by which NF-κB contributes to apoptosis is through transcriptional regulation of apoptotic gene targets. For example, the NF-κB mediated expression of TRAIL in T-cell leukemia is essential for apoptosis (Rivera-Walsh et al., 2001). The p65 subunit can bind the intronic region of genes transcribing DR5 and DR4, thereby inducing pro-apoptotic signaling (Ravi et al., 2001). Furthermore, inhibition of NF-κB by sodium salicylate can rescue cells from apoptosis in response to TRAIL (Rae et al., 2007). Another DR, Fas, has been implicated in NF-κB mediated apoptosis in adenovirus infected hepatocytes (Kuhnel et al., 2000) and in adenocarcinoma cells treated with TNF-α (Kimura et al., 2003). In glioblastoma cell lines, NF-κB exerts a pro-apoptotic function in TRAIL- or CD95-induced apoptosis, which can be reversed by over-expression of the dominant-negative IκBα-super-repressor (IκBα-SR). Conversely, stimulation of NF-κB, due to over-expression of constitutively active IKKβ, significantly increases TRAIL- or CD95-mediated apoptosis. In this instance it was found that NF-κB promotes the formation of the TRAIL or CD95 death-inducing signaling complexes (DISCs) through enhanced recruitment of FADD and caspase-8 to the activated receptors (Jennewein et al., 2012).

Unlike other NF-κB family members, NF-κB2/p100 is found to play a pro-apoptotic role, acting as both an activator of caspase-8 and as a IκB protein. The carboxyl terminus of p100 contains a death-domain, which mediates recruitment to and formation of DISCs. Tumor-derived p100 mutants often lack this domain, providing resistance from death receptor mediated apoptosis. HT1080 cells transfected with a p100 mutant containing only the death-domain underwent apoptosis. This pathway to apoptosis was found to be dependent on caspase-8 activation, as the caspase inhibitor CrmA negated the pro-apoptotic effect (Wang et al., 2002).

It has been shown in a p53-inducible cell line, Saos-2, that induction of p53 results in NF-κB activation and subsequent apoptosis. Inhibition or loss of NF-κB activity, abrogated p53-induced apoptosis, indicating that NF-κB is essential in p53-mediated cell death (Ryan et al., 2000). Similarly, in a study of murine fibroblasts, NF-κB was shown to mediate p53 activation, thereby inducing pro-apoptotic signaling (Fujioka et al., 2004). Not only can NF-κB result in p53 expression but a novel transcriptional target of NF-κB, polo-like kinase-3 (PlK3), was shown to phosphorylate p53 thus increasing its half-life and potency (Li et al., 2005b). Elevated expression of p65 alone can be sufficient for the induction of cell death, for example in MCF7/ADR breast cancer and M14 melanoma cells (Ricca et al., 2001). In these examples it is clear that NF-κB can play an important role in apoptotic signaling.

In the auditory cell line HEI-OCI, cisplatin induced both apoptosis and NF-κB signaling. Pre-treatment of HEI-OCI cells with the NF-κB inhibitor, Bay 11-7085, completely abrogated the apoptotic effects of cisplatin, indicating a pro-apoptotic role for NF-κB in this cell line (Chung et al., 2008). Site specific phosphorylation leads to differential expression of NF-κB target gene subsets, depending on the κB-response elements present at individual promoters (Anrather et al., 2005). In support of this, p65 induced in response to certain cytotoxic stimuli such as UV light and certain chemotherapeutic drugs is distinct from that induced by TNF-α. NF-κB induced in response to these stimuli is associated with histone deacetylases (HDACs) to suppress transcription of several anti-apoptotic genes, including Bcl-xL and XIAP (Campbell et al., 2004). It is therefore conceivable that post translational modifications of NF-κB subunits promotes differential responses by regulating interactions with promoters of transcription, such as histone acetyltransferases (HATs) and repressors, including HDACs (Miyamoto, 2004).

Chromatin remodeling controls the access of translational machinery and transcription factors to DNA, thereby affecting gene expression. Changes in histone acetylation status of NF-κB genes have been implicated in pro-apoptotic NF-κB signaling. In HEK293 cells, NF-κB can mediate cell survival through the EGFR but mediates apoptosis through Fas interactions (Gibson, 2004). In response to EGFR stimulation, NF-κB is recruited to both pro- and anti-apoptotic genes. At pro-apoptotic genes, NF-κB is complexed with HDACs causing transcriptional repression. In contrast, at anti-apoptotic gene sites, NF-κB is complexed with HATs causing transcriptional activation (Graham and Gibson, 2005). It is likely that several factors, including cell type, nature of stimulus, and chromatin modifications confer the pro or anti-apoptotic activities of NF-κB.

## Targeting NF-κB

A vast body of evidence exists to support the rationale of pursuing novel therapies which modulate the NF-kB pathway. Several points of therapeutic intervention have been proposed including IKK activation, IκB degradation, and NF-κB DNA binding (Nakanishi and Toi, 2005). However, due to its key role in a wide spectrum of cellular processes and its regulatory complexity, concerns have been raised over potential off-target effects and associated toxicities. It is only by fully elucidating the multiple tiers of NF-κB activation and its regulatory complexity in cancer that we can begin to design and select appropriate targeted therapies. One of the success stories in NF-κB targeting is the ubiquitin-proteasome inhibitor bortezomib, approved for clinical use in newly diagnosed and relapsed/refractory multiple myeloma and multiple mantle cell lymphoma patients (Kane et al., 2006). A proposed mechanism of bortezomib action is through the inhibition of IκB degradation resulting in the abolition of NF-κB signaling (Tan and Waldmann, 2002).

Following the success in hematological cancers, the efficacy of bortezomib therapy was investigated in solid tumors. Despite promising pre-clinical data, bortezomib in combination with temozolomide lacked efficacy in advanced refractory solid tumors or melanoma patients in a phase II clinical trial (Clinicaltrials.gov identifier:NCT00512798) (Amiri et al., 2004). Similarly, bortezomib in combination with other chemotherapeutic agents, including paclitaxel and carboplatin, failed to alter NF-κB expression, showed limited clinical advantage and significant toxicity in patients with advanced solid tumors (Croghan et al., 2010). Various studies with bortezomib in the treatment of advanced solid tumors, e.g., metastatic breast cancer (Yang et al., 2006a) and urothelial cancer (Gomez-Abuin et al., 2007), have proven disappointing. Numerous trials are currently being conducted to investigate the potential of bortezomib as an adjuvant to chemotherapy in head and neck (Fury et al., 2012) and NSCLC (NCT01633645). Bortezomib has previously shown a potential therapeutic benefit in NSCLC patients and acts synergistically with cisplatin-gemcitabine chemotherapy to constitute an active regimen in advanced stage NSCLC patients (Voortman et al., 2007).

Other compounds that may be useful in increasing the sensitivity of cancers with constitutively active NF-κB to chemotherapeutic drugs are herbs with anti-inflammatory properties including the natural phenol curcumin and parthenolide which occurs in the plant feverfew (Patel et al., 2000). Curcumin down-regulates transcription factors important for cell growth and survival, through modulation of the NF-κB and PI3K/AKT pathways (Reuter et al., 2008). Curcumin alone or in combination with chemotherapy is effective both *in vitro* and *in vivo* in a number of cancer types, including melanoma (Odot et al., 2004). The side effects of curcumin appear to be limited, with high oral doses being tolerated with minimal toxicity, although abdominal complaints have been reported (Lao et al., 2006; Epelbaum et al., 2010). A phase I clinical trial of patients with multiple myeloma showed that both curcumin alone or in combination with bioperine, an alkaloid isolate from black pepper, decreased NF-κB levels in peripheral blood mononuclear cells (PBMCs) (NCT00113841). Similarly, in a phase II trial, curcumin decreased NF-κB levels in PBMCs and possessed biological activity in some patients with advanced pancreatic cancer (Dhillon et al., 2008).

The aforementioned treatments are compounds which are not targeted NF-κB therapies. Recently, attention has shifted to therapeutic agents which selectively target NF-κB. One such compound, BMS-345541 selectively inhibits the catalytic subunit of IKK and can suppress tumor growth in murine melanoma models (Yang et al., 2006b). The cell permeable NBD (NEMO-binding domain) peptide prevents NF-κB activation by binding NEMO, thereby inhibiting NEMO-IκB complex formation (di Meglio et al., 2005). *In vitro* studies demonstrate that NBD impairs the ability of NF-κB to bind DNA, resulting in increased apoptosis in melanoma cells (Ianaro et al., 2009). The NF-κB specific inhibitor DHMEQ, prevents the nuclear translocation of the transcription factor, and has been shown to have anti-cancer effects in numerous different cancer subtypes (Umezawa and Chaicharoenpong, 2002; Umezawa, 2006). In addition, the testing of small molecule inhibitors to neutralize NF-κB have identified potential new cancer therapeutics, which require further investigation as to their clinical benefit (Miller et al., 2010; Hwang et al., 2012; Mora et al., 2012). The small molecule inhibitor, bindarit, an indazolic derivative, which may down-regulate NF-κB through reduced phosphorylation of IκBα and p65 (Zollo et al., 2012), has subsequently been shown to modulate cancer cell proliferation and migration, impairing metastatic disease in murine models of prostate and breast cancer, through NF-κB and AKT inhibition (Zollo et al., 2012).

## Conclusion

Aberrant NF-κB expression and regulation is involved in the development of many different cancer types, where it mediates the fine balance between cellular survival and death. The importance of NF-κB activation in cancer cells is evidenced by the widespread dysregulation of this transcription factor across a wide spectrum of solid and hematological cancers. NF-κB is also heavily implicated in the development of resistance to platinum-based chemotherapies, such as cisplatin. Therefore, NF-κB represents an attractive target for anti-cancer therapy particularly as an adjuvant to overcome resistance to platinum-based chemotherapeutics.

The efficacy of targeted therapy is based on the oncogenic dependence of cancer cells on mutated survival and apoptotic pathways, such as NF-κB, which renders them more susceptible to inhibitors. NF-κB is a master regulator of transcription and can affect the expression of over 200 genes. Due to its overwhelming influence on cellular processes, care must be exercised when blocking the NF-κB pathway to minimize off-target effects and unwanted toxicities. Furthermore, as NF-κB is a converging point for many intercellular pathways, it may be difficult to identify the most effective pathway to target. Elucidation of cell specific pathways of NF-κB activation, as well as oncogene and apoptotic status, could provide the means to effectively and specifically target fragments of the NF-κB signaling network. Indeed it may prove more beneficial to target a number of regulators of this pathway through combinatorial therapeutic strategies. This may not only aid in cancer diagnosis and prognosis but may also provide an effective avenue to re-sensitize cancers to cisplatin based therapies.

## Conflict of Interest Statement

The authors declare that the research was conducted in the absence of any commercial or financial relationships that could be construed as a potential conflict of interest.
